# On the aerodynamics of dual-stage co-axial vertical-axis wind turbines

**DOI:** 10.1177/0309524X231212638

**Published:** 2023-12-09

**Authors:** Muhammad Saif Ullah Khalid, Priscila Scarlet Portocarrero Mendoza, David Wood, Arman Hemmati

**Affiliations:** 1 Department of Mechanical and Mechatronics Engineering, Lakehead University, Thunder Bay, ON, Canada; 2 Department of Mechanical Engineering, University of Alberta, Edmonton, AB, Canada; 3 Department of Mechanical and Manufacturing Engineering, University of Calgary, Calgary, AB, Canada

**Keywords:** Multi-stage wind turbines, bio-inspired engineering, computational fluid dynamics

## Abstract

This study explored the aerodynamics of a new multi-stage co-axial vertical-axis wind turbine based on bio-inspiration from natural swimming habit of fish. The turbine was formed from a conventional straight-bladed vertical axis turbine (VAWT) with an additional small inner rotor, also of three blades. The azimuthal and radial locations of the inner rotor were varied. Using numerical simulations, performance of the proposed new design was evaluated over a range of tip-speed ratios. The preliminary results identified a 600% increase in power output for multi-stage VAWTs at tip-speed ratios TSR<3 , and a substantial drop in power coefficient at TSR>3.0 . The wake dynamics analyses revealed that the increase was due to interactions between the blades of one rotor and the other. This reduced the unsteady separation from the outer rotor, which produced most of the power. A detailed parametric study was also completed, which showed the implications of geometric and kinematic details on the performance of the proposed multistage VAWT.

## Introduction

Energy is an essential commodity in defining our future prosperity. The scarcity of traditional resources and their limited sustainability, combined with their substantial contributions to climate change, have motivated extensive investment in research and development of alternative energy technologies. In this context, wind provides a great source of energy that is harnessed by different types of turbines. Vertical-axis wind turbines (VAWTs) are considered a great choice for energy harvesting in urban settings for small-scale power generation at lower wind speeds. Nevertheless, these turbines are also less efficient compared to horizontal-axis wind turbines (HAWTs) due to fluctuating aerodynamic loads, the consequent fatigue issues (Galinos et al., 2016), and continuous variations in the angle-of-attack ( α ) for their blades with respect to the on-coming wind (Bazilevs et al., 2014). These features contribute to more complex aerodynamics of VAWTs. Moreover, VAWTs are categorized into Savonius, Darrieus, and H-rotor types. Eriksson et al. (2008) presented a comparison of these turbines and argued that H-rotor type VAWTs offer more advantages in comparison to the other versions, including their simpler structures and no requirements of pitch regulators, yaw mechanisms, or gearboxes. However, these turbines also suffer from poor self-starting capabilities (Asr et al., 2016; Hill et al., 2009; Sun et al., 2021).

In order to increase the power output of VAWTs, various design modifications were proposed over the years. A popular strategy is to install VAWTs in parallel (Dabiri, 2011; Hassanpour and Azadani, 2021; Lam and Peng, 2017a; Li et al., 2021; Peng et al., 2020; Zanforlin and Nishino, 2016) or tandem configurations (Ni et al., 2021; Sahebzadeh et al., 2020). Dabiri (2011) reported experimental investigations with VAWTs in counter-rotating arrangements and determined that closely spaced turbines could attain higher power densities, even greater than those for HAWTs, by efficiently extracting energy from adjacent wakes. Through their two-dimensional numerical simulations, Zanforlin and Nishino (2016) found that the presence of another turbine in the vicinity of a VAWT in a side-by-side configuration modified the direction of on-coming wind in such a manner that its lateral velocity component favored the production of greater lift and torque. Similarly, Lam and Peng (2017a) conducted flow measurements in a wind tunnel for co-rotating and counter-rotating VAWTs and concluded that counter-rotating turbines helped maintain flow symmetry in the wake. In their case, staggered arrangements of turbines also showed small wake spreading rates and rapid wake recovery, which is advantageous in designing wind clusters and farms. Later, they also presented that power extraction capability of twin turbines was 8% – 13% greater than that of a solitary VAWT (Lam and Peng, 2017b). Recently, Sahebzadeh et al. (2020) carried out three-dimensional simulations to find that an optimal spacing between two staggered rotors helped form a narrow region of high-speed flow in between them, which can increase the power coefficient of turbines installed in the downstream direction. Moreover, Hassanpour and Azadani (2021) showed that in order to maximize the performance of staggered arrays, VAWTs should be positioned at the same height from the ground. Otherwise, differences in heights reduced their power production.

Some researchers (Bhuyan and Biswas, 2014; Ghosh et al., 2015; Kumbernuss et al., 2012) experimented with hybrid models of VAWTs. To improve the self-starting capability of VAWTs, Bhuyan and Biswas (2014) proposed a novel design by placing a Savonius rotors on top of a 3-bladed H-type rotor. This hybrid design was able to self-start for all azimuthal angles by producing positive static torque. However, its power coefficient ( $C_{P}$ ) depended on extent of the overlap of its two parts. The maximum value reached 0.34 , before dropping down due to further increase in overlap at tip-speed ratio ( λ ) of 2.29 and Reynolds number ( Re ) of $1.92\times 10^{5}$ . The power output of each turbine was higher than the output of a single turbine. A similar approach was utilized by Ghosh et al. (2015) to design a combined 3-bladed Darrieus-Savonius wind turbine for low- Re flows in built environments. Also, Kumbernuss et al. (2012) employed two counterrotating Savonius turbines to develop a double stage VAWT with one stage directly above the other. The phase shift angle between the two stages in relation to wind speeds affected its performance significantly. More stages were added in this design of multi-stage Savonius turbine by Saad et al. (2021). It was reported that the maximum $C_{P}$ was 0.253 and 0.261 for two- and four-stage rotors, respectively, whereas a single-stage rotor obtained $C_{P}=0.223$ . The greater advantage of the multistage systems was the reduction of oscillations in torque and thrust during rotational cycles, which was expected to mitigate fatigue and associate flow-induced noise, while improving the structural integrity of the system. This concept was also extended to H-rotor type VAWTs by Didane et al. (2018, 2019), where two 3-bladed rotors were installed together in co-axial contra-rotating settings. This configuration enabled tripling $C_{P}$ and torque compared to a single-stage turbine (Didane et al., 2018). Next, they performed three-dimensional numerical parametric studies (Didane et al., 2019) and found that keeping those two co-axial rotors close to each other increased their power output significantly.

New designs of VAWTs can also be developed by installing rotors in series arrangements (Arpino et al., 2017, 2018, 2020; Malael and Dragan, 2018; Scungio et al., 2016; Su et al., 2020; Tahani et al., 2020; Torabi Asr et al., 2016; ZSB-AB, 2011). The first attempt with this approach involved two co-axial coupled rotors installed in series (ZSB-AB, 2011). Both outer and inner rotors contained 3 blades and the inner one rotated in the envelop of the outer rotor. Later, Torabi Asr et al. (2016) performed two-dimensional simulations for the flow-induced rotation of coupled and uncoupled versions of these multi-stage VAWTs. Their results indicated that the presence of another rotor inside the primary one accelerated within smaller periods of time, which made it applicable to low-speed wind turbines. Moreover, Malael and Dragan (2018) and Tahani et al. (2020) also employed similar configurations of two rotors in counter-rotating and co-rotating arrangements to explain their underlying flow dynamics. Tahani et al. (2020) determined that the additional inner stage of the VAWT enhanced $C_{m}$ and $C_{P}$ by more than 300%. Perhaps, the most concerted efforts in this subject were carried out and reported by Scungio et al. (2016) and Arpino et al. (2017, 2018, 2020). First, Scungio et al. (2016) showed that having three pairs of main and auxiliary blades instead of a conventional H-rotor type VAWT produced more dynamic torque for a large range of wind speeds. It also reduced the time taken to start the turbine from rest. Arpino et al. (2018) performed extensive wind tunnel testing and numerical simulations to demonstrate that their multi-stage turbine was able to harness sufficient energy for wind speeds lower than 4m/sec . Recently, Su et al. (2020) modified this design and found that connecting the auxiliary blades with leading edges of the main blades, and pitching them inwards, helped control power output of the turbine more effectively for varying wind speeds.

These studies revealed great potential for improvements in currently available designs of VAWTs to enhance energy harvesting from natural winds in urban environments. Hybrid and multi-stage turbines remain the subject of extensive research. However, our understanding about these innovative systems is limited and more efforts are required to explain their underlying governing aerodynamic mechanisms. In this quest, we drew our inspiration from fish schooling phenomena to propose a new design of VAWTs with increased energy harvesting capabilities and better aerodynamic performance. It is well known (Gungor and Hemmati, 2020, 2021; Khalid et al., 2016) that fish utilize specific configurations that enable significant advantages by harnessing more energy from the vortices generated by other fish in their vicinity. They tend to form various configurations to perform different social and hydrodynamic functions. These include circular arrangements, where individual members of schools position themselves in the form of co-axial circular loops with different radii, which resemble turbines from a two-dimensional perspective. Pan and Dong (2020) demonstrated that dense arrangements of the members in a fish school was more advantageous for their hydrodynamic performance. In the context of modern-day technology, lessons learnt from natural schooling phenomena can also provide potentially effective solutions for various problems related to energy harvesting through tidal (Kinsey and Dumas, 2012) and wind turbines (Brownstein et al., 2016; Kinzel et al., 2012). Hence, we employed these ideas to computationally investigate dual-stage co-axial turbines in more detail. Moreover, we explained the effects of various geometric and kinematic parameters on their performance and flow features. Recently, we also presented a detailed analyses on the self-starting response of these dual-stage turbines in comparison to that of a single-stage one (Khalid et al., 2022). However, the present manuscript addresses the important aspect of the power production capacity of these VAWTs designs for a wide range of important design parameters, including tip-speed ratios, ratio of the radii for the two rotors, and their relative azimuthal positioning, as explained in Table 1.

**Table 1.** Flow parameters and geometric characteristics of VAWTs, where “1” represents the primary, outer rotor and “2” the inner, secondary one, based on reference (Khalid et al., 2022).

Parameters	Value
Airfoil section	NACA0018
Number of Blades in single-stage turbines	3
Number of Blades in dual-stage turbines	6
Tip-speed ratio ( TSR )	1.50−4.50
Free-stream velocity ( $U_{\infty }$ )	7m/s
Chord length of blades in rotor 1 ( $c_{1}$ )	0.06m
Radius of rotors 1 ( $R_{1}$ )	0.5m
Rotors 1 and 2 ratios of radii	$R_{1}/R_{2}=0.85$ & 0.92
Geometric ratios for blades in rotors 1 and 2	$c_{1}/D_{1}=c_{2}/D_{2}=0.06$
Angle between the blades of rotors 1 and 2 ( ϕ )	0° – 90°
Area ( A )	$1m^{2}$

This study is organized as follows. Section 2 elucidates our computational methodology to perform simulations for single-stage and dual-stage VAWTs. Next, our findings on the performance of these VAWTs and their aerodynamic mechanisms are illustrated in section 3. Lastly, the summary and conclusions are presented in section 4.

## Numerical methodology

The computational methodology used to perform simulations for flows over rotating turbines with prescribed angular velocities is described in this section. It includes discussions of the kinematics and details of the numerical setup, which is followed by detailed verification and validation studies.

## Geometry and kinematics

**Figure 1.**
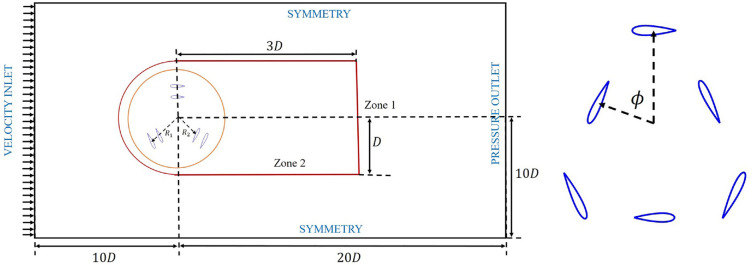
A schematic representation of the computational domain with details on boundary conditions.

This research employed vertical-axis wind turbines of the H-type Darrieus model with NACA0018 symmetric airfoils, both single-stage and dual-stage. These airfoils depicted the blade’s cross-section. A visual representation of the turbines, including important kinematic and geometric information, can be found in Figure 1. Table 1 provides additional details on governing parameters used to assess the performance of the turbines.

Usually, the flow, geometric, and kinematic characteristics of wind turbines are described using the following parameters. The first parameter is the tip-speed ratio, which is defined as:

(1) $$\begin{array}{c} TSR=\frac{\Omega R_{1}}{U_{\infty }} \end{array}$$

where $R_{1}$ denotes the radius of the primary rotor, Ω is the angular velocity, and $U_{\infty }$ represents the free-stream flow velocity. We tested the performance of VAWTs for TSR , ranging from 1.50to4.50 , covering a broad range for wind turbine operation. For these kinematic conditions, Rezaeiha et al. (2017a) showed that the results from 2D simulations approached those from 2.5D simulations.

## Flow solver

In our present work, ANSYS Fluent 2020R2 (ANSYS, 2020) was used to perform simulations. This solver has gained a lot of popularity among researchers for wind turbine simulations (Ghasemian and Nejat, 2015; Mohamed, 2014, 2016; Siddiqui et al., 2015). Incompressible unsteady Reynolds-averaged Navier-Stokes (URANS) and continuity equations were solved in Cartesian coordinates through the pressure-based solver:

(2) $$\frac{\partial u_{j}}{\partial x_{j}}=0$$

(3) $$\frac{\partial u_{i}}{\partial t}+\frac{\partial }{\partial x_{j}}\left(u_{i}u_{j}\right)=-\frac{1}{\rho }\frac{\partial p}{\partial x_{i}}+\nu \frac{\partial^{2}u_{i}}{\partial x_{j}\partial x_{j}}$$

where $x_{j}$ denotes the Cartesian coordinates, and j={1,2} . Here, u is the Cartesian velocity components, ρ is the fluid density, p is the pressure, and ν indicates the kinematic viscosity. The Pressure Implicit with Splitting Operators (PISO) scheme is commonly suggested for unsteady flows (Thé and Yu, 2017) and offers benefits when using large time-steps to advance in time during computations. Therefore, to enhance computational efficiency, the Semi-Implicit Method for Pressure Linked Equation Consistent (SIMPLEC) algorithm was employed.

For computation of gradient terms, the least square cell-based technique was used. Convective pressure terms and diffusion terms in momentum equation (equation (3)) were approximated with second-order scheme and second-order upwind scheme, respectively. Third-order scheme may also be used for Laplacian operator terms. However, those are computationally expensive. Nevertheless, the upwind scheme provides greater stability in numerical simulations. The second-order implicit scheme was utilized for the unsteady term.

First, the moving reference frame ( MRF ) approach was used to obtain steady-state flow features around the turbine, where the turbine did not actually rotate. The results from this analysis were then used as the initial condition to carry out unsteady simulations. This approach helped attain faster convergence of iterative solutions at each time-step. The sliding mesh technique was used to perform unsteady simulations. In these areas, the physical rotation of the turbine was permitted without causing any disruptions to the original mesh.

Rezaeiha et al. (2019) recommended the use of the Shear Stress Transport (SST) turbulence models for accurately capturing flow characteristics in VAWTs, as their findings were closely aligned with experimental results. Therefore, the 4-Equations SST- k - ω model was employed to forecast turbulent flow characteristics. Developed by Menter (1994), this model combines the robustness and accuracy of the k - ω model in near-wall regions with free-stream independence of the k - ϵ model in the far field. This blended formulation refines the standard k - ω model by modifying the definition of turbulent viscosity to incorporate the transport of turbulent shear stress. It enhances the accuracy and reliability of turbulence modeling for a wide range of flows with adverse pressure gradients.

The convergence criterion at each time-step was set to $10^{-4}$ . Although we obtained convergence within 10−15 iterations at each time-step, maximum allowable number of iterations were 50 . All the simulations were completed for 22 revolutions, which provided the statistical quantities based on the data of last 5 revolutions. As also discussed by Rezaeiha et al. (2017a), the steady-state solutions were achieved within 15−20 revolutions of the turbine.

## Computational domain and boundary conditions

A rectangular computational domain with the H-grid technique was used in this study, which is shown in Figure 1. The inlet boundary situated at a distance of 10D from the turbine axis was prescribed with a uniform flow velocity ( $U_{\infty }=7m/\sec$ ). The pressure outlet boundary was set as the zero gauge pressure, located 20D from the rotational axis. The top and lower boundaries were designated as symmetry boundaries and were positioned at a distance of 10D from the turbine axis, respectively. All boundaries within the domain were established based on the guidelines provided by Rezaeiha et al. (2017a).

**Figure 2.**
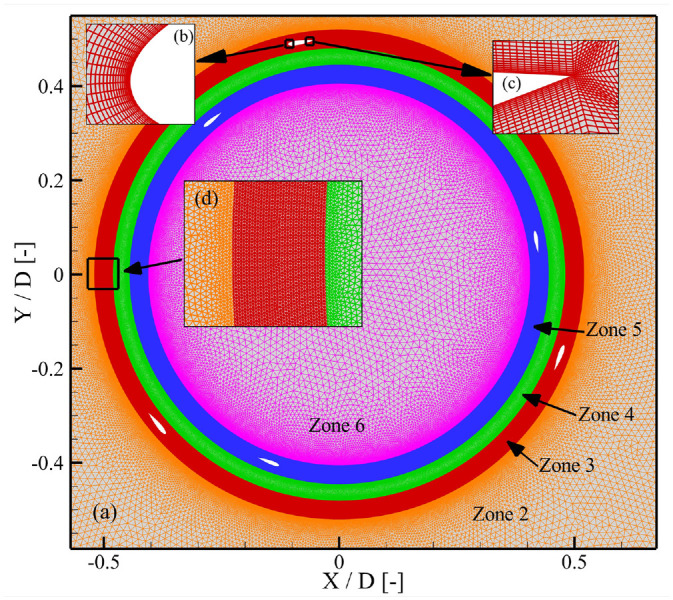
Mesh settings in different zones of the flow domain and near the blades: (a) inside the rotating domain and around it, (b) near the leading edge, (c) close to the trailing edge, and (d) variation of mesh around interfaces between zone 4, zone 5, and the stationary domain.

Five zones for dual-stage VAWTs were defined in the computational domain to incorporate the moving reference frame and sliding mesh techniques. In Figure 1, zone 1 and zone 2 are shown and both of them remained stationary. The neighboring domains in this study were connected through an interface that facilitated the exchange of flow information, using a non-conformal meshing algorithm. The flow characteristics in the wake of the rotating turbine were captured in Zone 2. Inside zones 3–6, as shown in Figure 2(a), the meshing features were depicted. Zone 3 housed the blades for the outer stage of the turbine, while Zone 5 contained the blades for the inner stage. These domains rotated around the central axis of the vertical-axis wind turbine (VAWT). However, the domains represented by zones 4 and 6 remained stationary. Figure 2(b) and (c) displayed the grid near the leading and trailing edges of the airfoils, respectively, providing sufficient accuracy in capturing the boundary layer around these rotating structures. Mesh settings were controlled to slowly change the mesh size and avoid large gradients, as shown in Figure 2(d). To mitigate numerical errors, a uniform cell size was maintained around the interface boundary separating the rotating and static domains. This setup followed the recommendations of Rezaeiha et al. (2017a). It revealed that radius of a rotating domain had no significant implication on aerodynamics of wind turbines in simulations.

## Performance parameters

Nondimensional power and torque coefficients, denoted as $C_{P}$ and $C_{T}$ , respectively, were calculated for every case, to measure aerodynamic performance. These were defined as follows:

$$\begin{array}{cc} C_{T}=\frac{T}{qA}, & C_{P}=\frac{T\Omega }{qU_{\infty }A} \end{array}$$

where T represents torque of the turbine, A represents the swept area of the turbine, and $q=\rho {U^{2}}_{\infty }/2$ , the dynamic pressure. Swept area was a factor calculated through multiplication of the turbine height ( 1m for the current 2D cases) and its outer diameter. Using time-period ( τ=1/f ) of one revolution, corresponding time-averaged coefficients were computed using the following relation:

$$C=\frac{1}{\tau }\int_{t}^{t+\tau }C\left(t\right)dt$$

Steady-state power coefficient was calculated using the relation $\bar{C_{P}}=(\mathrm{TSR})\;\bar{C_{T}}$ . The statistical quantities were computed for the last 5 revolutions of the VAWTs, where the variations in the time-averaged quantities were negligible (Rezaeiha et al., 2017a).

## Grid independence study

A detailed sensitivity study was completed to ensure grid convergence for the dual-stage VAWTs. Unstructured triangular cells in the fluid domain were used with quadrilateral elements were arranged in 26 layers around each blade to accurately address the boundary layer. The grid size was adjusted by varying the maximum sizes of the grid in different zones, ensuring that the value of $y^{+}$ remained approximately 1. It helped estimate the first cell height from the solid surface. Its accuracy is important to resolve the viscous sublayer in turbulent boundary layers. To carry out verification of the simulations results, three mesh sizes were considered for λ=4.5 , whose details are shown in Table 2.

**Table 2.** Mesh details in different zones for grid-independence study based on reference (Khalid et al., 2022).

Grid details	G1 coarse	G2 medium	G3 fine
Mesh nodes on each blade	400	400	400
Maximum size in zones 3 and 5	0.002	0.00135	0.001
Maximum size in zone 4	0.0065	0.0575	0.005
Maximum size in zones 6 and 2	0.02	0.015	0.01
Total quantity of cells	366,164	600,721	1,015,919

**Figure 3.**
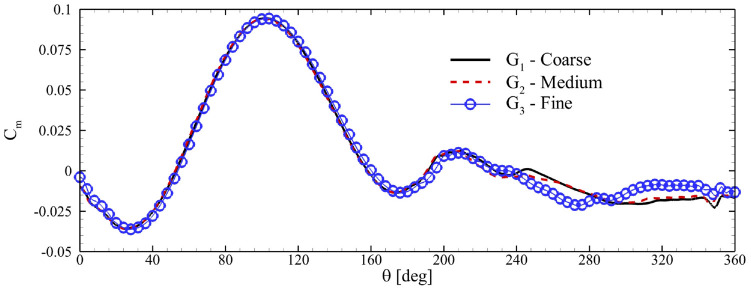
$C_{m}$ of a single blade for different grid configurations.

Variations in the moment coefficient of one blade in a dual-stage rotor, undergoing rotation with λ=4.0 . are depicted in Figure 3. Apparently, three meshes produced the same $C_{m}$ and small differences were presented only for 240°<θ<350° . The following simulations were conducted using the mesh settings of $G_{2}$ with the change of the azimuth angle dθ=0.2° at each time-step (Rezaeiha et al., 2017a, 2017b) since grid configuration $G_{2}$ exhibited a closer resemblance to $G_{3}$ . Readers are referred to Khalid et al. (2022) for more details about the verification of our simulation methodology.

## Validation

**Figure 4.**
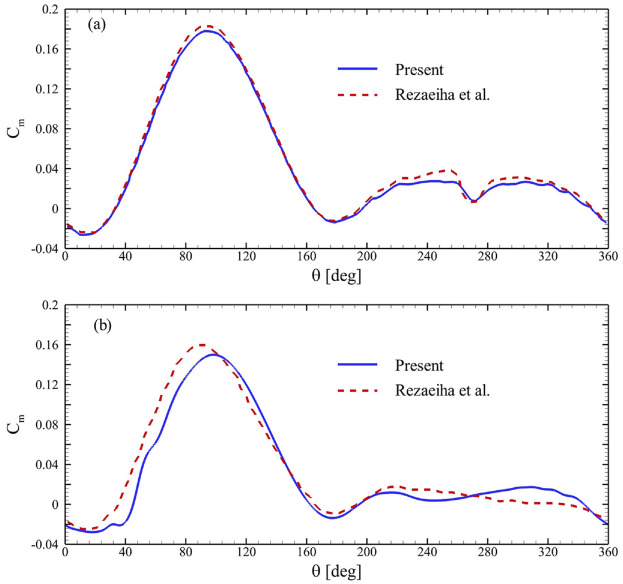
Comparison of Cm with those from numerical simulations of flows around a (a) 2-bladed VAWT (Rezaeiha et al., 2017b) and (b) 3-bladed turbine (Rezaeiha et al., 2017a).

To the best of the authors’ knowledge, there are no experimental data in literature corresponding to the co-axial and co-rotating VAWTs (Lam and Peng, 2017a; Naccache and Paraschivoiu, 2018; Vergaerde et al., 2020). There exist designs composed of two VAWTs in different arrangements. However, the proposed VAWT design is inherently different from those systems that have been reported in literature. In order to maintain consistency in literature, we chose to validate our simulation methodology through the available data of a single-rotor turbine. The simulations were validated by initially comparing their results with those reported in Rezaeiha et al. (2017a) and Rezaeiha et al. (2017b), for 2-bladed and 3-bladed single-stage turbines, respectively. Figure 4(a) exhibits $C_{m}$ of a single blade in a 2-bladed VAWT obtained by the present simulation settings and those of Rezaeiha et al. (2017a). It is evident that both profiles matched well for the complete rotation of the blade in Figure 4. Additionally, $C_{m}$ followed similar trends for the 3-bladed VAWT with minimal differences in magnitude. Moreover, simulations for VAWTs used by Castelli et al. (2011) were repeated to compare the computational results with their experimental and numerical results. Table 3 shows that the current numerical values of $C_{P}$ were closer to the experimental measurements for low values of tip-speed ratios. The difference between the numerical and experimental values were caused due to several reasons, including the absence of three-dimensional flows around the blades’ tips and geometric features, such as spokes, connecting struts, and the central shaft. At low tip-speed ratios, dynamic stall phenomenon could also have played a major role the association of which with the absence of afore-mentioned structural elements caused an overestimation of $C_{P}$ . It is also important to mention that Castelli et al. (2011) did not give the details of turbulence intensity and other relevant parameters to handle the associated uncertainties. In the present simulations, a turbulence intensity of 3% was used. Nevertheless, the present solver performed better and our values of $C_{P}$ were found closer to the experimental ones.

**Table 3.** Comparison of the $C_{p}$ from present results with those from literature.

TSR	Exp. (Castelli et al., 2011)	CFD (Castelli et al., 2011)	Present
1.44	0.013	0.17	0.055
1.68	0.044	0.251	0.103
2.04	0.138	0.431	0.172

## Effect of turbulence models

**Figure 5.**
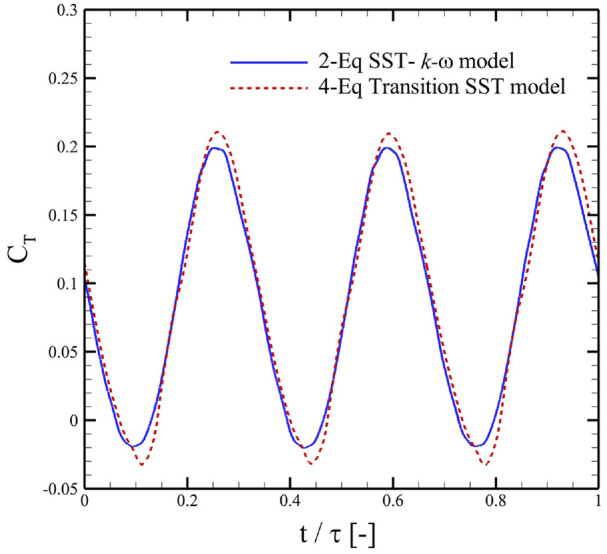
Comparison of the torque coefficients ( $C_{T}$ ) computed using two-equations SST-k-w model and 4-equations transition SST model for flows over a multistage VAWT with ϕ=0° and $R_{2}/R_{1}=0.85$ at TRS=3.0.

In order to demonstrate the suitability of the turbulence model, a comparison of steady-state torque coefficients ( $C_{T}$ ) for the whole turbine around its central axis computed through 2-equations SST- k - ω and 4-equations transition SST models was presented in Figure 5. Here, τ denoted the time-period for a complete revolution of the turbine. The plots clearly showed that the torque profiles remained largely unaffected by the choice of the turbulence model. The only minimal difference was observed at the time instants when $C_{T}$ attained its maximum and minimum values. Previously, Rezaeiha et al. (2019) presented a detailed analysis of the effectiveness for difference turbulence models. They found that the employment of transition SST models captured the laminar-to-turbulence transition quite well. However, the results of all SST-based turbulence models matched closely with those obtained through experiments for a wide range of flow and kinematic governing parameters. Because the employment of 3-equations and 4-equations model are known to increase the computational cost by 14% and 30%, respectively (Rezaeiha et al., 2019), we chose the 2-equations SST- k - ω model to proceed with the remaining simulations.

## Results and discussion

**Figure 6.**
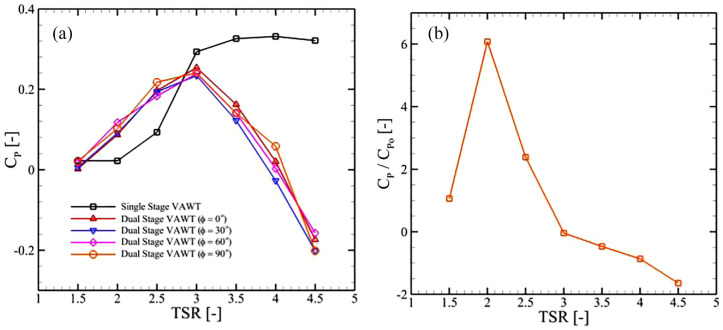
(a) Variations in $C_{P\;}$ as a function of TSR for the single-stage turbine and dual-stage VAWTs ( $R_{2}/R_{1}=0.85$ ) for different values of ϕ and (b) ratio of power coefficients for a dual-stage turbine ( $R_{2}/R_{1}=0.85$ , ϕ=0° ) and the single-stage VAWT.

We begin by looking at the aerodynamic performance parameters and flow analysis around dual-stage VAWTs in comparison to those for their single-stage counterparts. Here, λ ranged from 1.50 to 4.50 , whereas the geometric phase angle between the outer and inner rotors varied from 0° to 90° . First, we presented a plot of $\bar{C_{P}}$ versus TSR in Figure 6(a). It is apparent that the single-stage VAWT produced similar power for TSR of 1.50 and 2.0 . It enhanced with further increase in TSR and reached its maxima for TSR=4.0 . However, the difference in $\bar{C_{P}}$ was minimal for 3.50<TSR<4.50 . On the contrary, the dual-stage VAWTs performed significantly better than the single-stage turbine for 1.50<TSR<3.0 . Our data showed that introduction of the secondary rotor improved the power production by up to 400% for this range of tip-speed ratios. These results also show that variations in ϕ did not substantially impact the energy harvesting capacity of dual-stage VAWTs and the trend for 0°<ϕ<90° remained the same. There were also negligible variations in $C_{P}$ . However, the dual-stage VAWTs suffered from sharp decrements in $C_{P}$ for TSR≥3.0 . This aspect of dual-stage turbines needed further investigations with variations in other important geometric parameters, including blades profiles and their aspect ratios.

Upon increasing the ratio between the radii of primary (outer) and secondary (inner) rotors by bringing the inner blades closer to the outer ones, a substantial improvement in the performance of the dual-stage turbine was achieved for low tip-speed ratio as evident by the plot in Figure 6(b). Here, the ratio of $C_{P}$ to $C_{\mathrm{Po}}$ was plotted, where $C_{P}$ belongs to a dual-stage turbine with $R_{2}/R_{1}=0.92$ and ϕ=0° and $C_{\mathrm{Po}}$ to a single-stage turbine. It showed improvements in power production by 600% for TSR=2.5 and 225% for TSR=3.0 by the dual-stage turbine. However, it was apparent from these results that $R_{2}/R_{1}=0.92$ did not suit the performance of the VAWT at higher TSRs .

**Figure 7.**
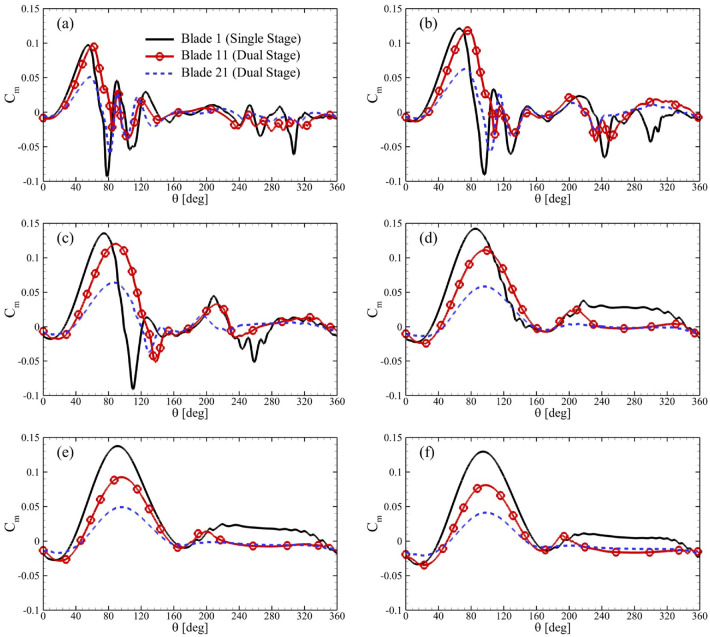
Variations in $C_{m}$ for single blades of the single-stage turbine, and outer and inner rotors of dual-stage VAWTs ( $R_{2}/R_{1}=0.85$ ) for ϕ=0° and TSR = (a) 1.50, (b) 2, (c) 2.50, (d) 3.0, (e) 3.50, and (f) 4.0.

In order to look deeper into the unsteady performance of these turbines, $C_{m}$ of single blades of the single-stage turbine were analyzed against primary and secondary rotors of dual-stage turbines in Figure 7. It is important to highlight that the results were presented on the same scale so that the effect of TSR could be demonstrated on the performance of these turbines. Here, the focus was on the performance parameters of the dual-stage VAWTs with ϕ=0° due to insignificant effect of ϕ on their $\bar{C_{P}}$ . The most important feature of this performance data was that blades in both stages of the dual-stage VAWT attained their maximum respective $C_{m}$ with specific delays for all TSR in comparison to the one in the single-stage turbine. Here, Blade 11 and Blade 21 were used for single blades in the outer and inner rotors of the dual-stage VAWT. For TSR=1.50 in Figure 7(a), Blade 1 appeared to reach its maximum $C_{m}$ for θ=54° . However, Blades 11 and 21 attained this state at θ=60° . This implied that adding an auxiliary rotor helped delay the dynamic stall process, which was most likely induced by the ground effect provided by blades in the inner stage of the VAWT. The next important point involved avoiding large negative values of $C_{m}$ by Blades 11 and 21 at θ=80° . It was evident that the blades of dual-stage VAWTs showed positive $C_{m}$ for a greater range of θ , which demonstrated the possibilities of better self-starting capabilities of multi-stage turbines. Besides, Blades 11 and 21 experienced significant reduction in magnitudes of their respective $C_{m}$ for TSR>3.0 as is also shown in their $\bar{C_{P}}$ in Figure 6. A plausible reason for better performance of the single-stage VAWT for higher TSR can be that its blades stopped producing negative $C_{m}$ for θ>100° , while there were negative values observed for a single stage system at similar conditions.

**Figure 8.**
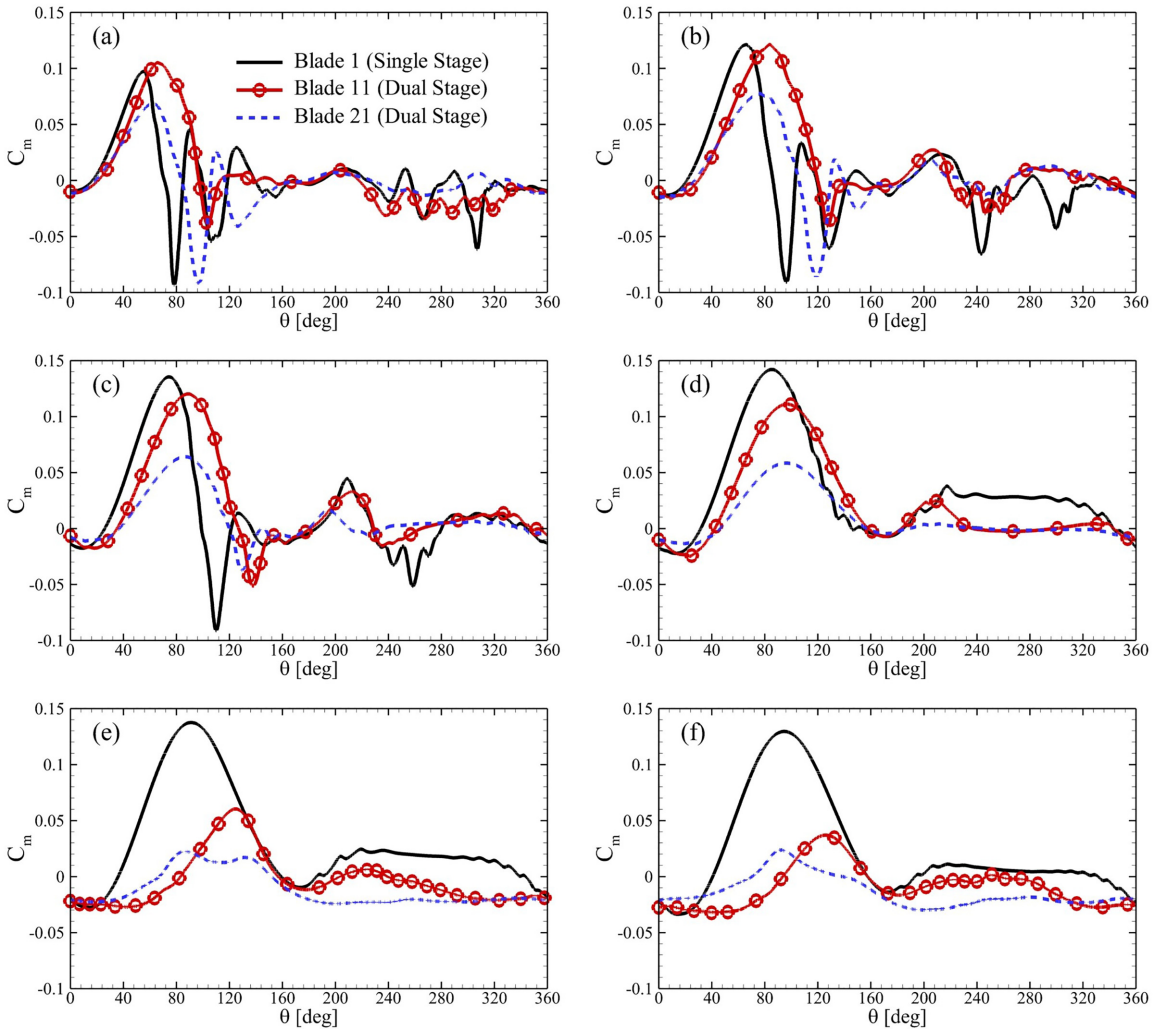
Variations in $C_{m}$ for single blades of the single-stage turbine, and outer and inner rotors of dual-stage VAWTs ( $R_{2}/R_{1}=0.85$ ) for ϕ=0° and TSR = (a) 1.50, (b) 2, (c) 2.50, (d) 3.0, (e) 3.50, and (f) 4.0.

Moreover, Figure 8 presents temporal profiles of moment coefficients of single-stage and dual-stage ( $R_{2}/R_{1}=0.92$ and ϕ=0° ) VAWTs. Here, Blade 11 exhibited a higher $C_{m}$ than Blade 1 at a greater azimuthal angle (see Figure 8(a)). For this blade of the primary rotor, negative value of $C_{m}$ was also significantly reduced. Despite these improvements, Blade 21 suffered from a negative $C_{m}$ with magnitudes that almost matched the order of Blade 1, contrary to what we observed for the inner rotor blade for the VAWT with $R_{2}/R_{1}=0.85$ in Figure 7(a). It is also important to highlight that $C_{m}$ of Blade 21 was lower than that of Blade 1 mostly for θ>200° at TSR=1.50 . However, it showed improvements in $C_{m}$ for these azimuthal positions at TSR=2.0 and 2.50 in Figure 8(a) and (b), respectively. The blades of the dual-stage turbine started showing signs that implied their reduced performance from TSR=3.00 in Figure 8(d) and both rotors individually produced less power compared to the single-stage version for almost the whole range of θ . It was even deteriorated for TSR≥3.50 .

**Figure 9.**
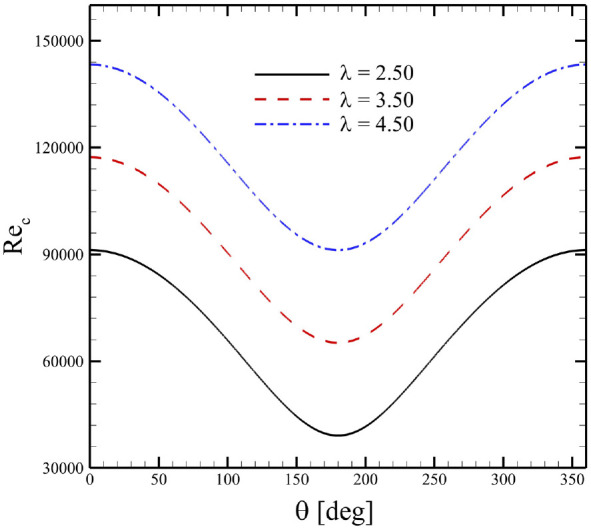
Variations in chord-based Reynolds numbers with respect to azimuthal positions.

This behavior of single- and double-stage VAWTs can be further elucidated by examining their production of lift and drag with respect to effective angles-of-attack of the blades. For this purpose, variations in Reynolds numbers throughout their rotational cycles were shown for different TSRs in Figure 9. Because the effective velocity of blades; a function of their azimuthal positions, was employed as the velocity scale for the computation of Re , its variation follows a cosine function when plotted against θ . It was evident that for each TSR , the difference in the maximum and minimum Re was around 40,000 . It showed extreme unsteadiness experienced by these blades during their rotation. To avoid this complexity, an average Re was chosen for further comparative analysis for aerodynamics of rotating turbines and static airfoils.

**Figure 10.**
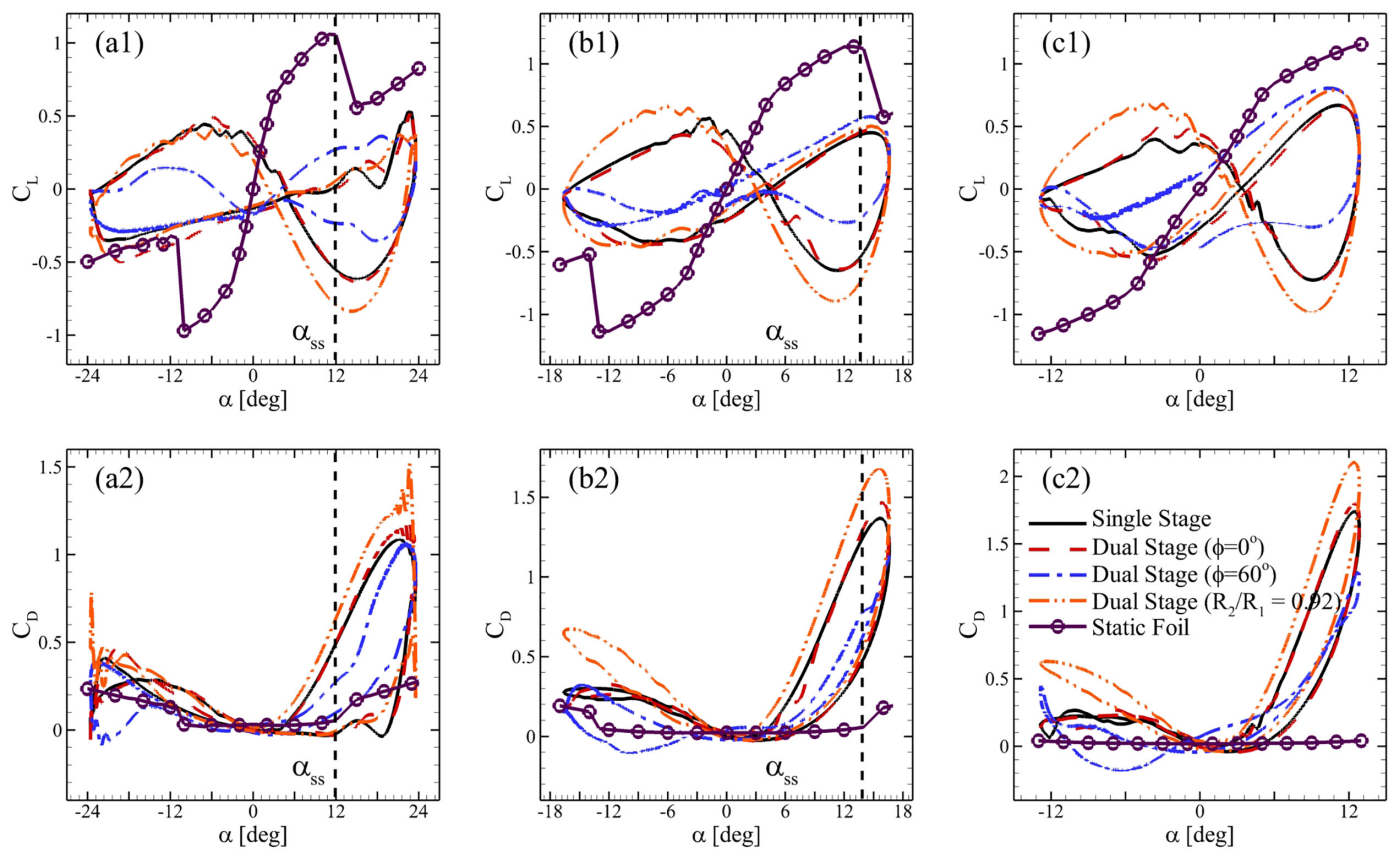
Variations of dynamic loads on a single blade with respect to α where (a1) and (a2) correspond to TSR = 2.50, (b1) and (b2) are for TSR = 3.50, and (c1) and (c2) present data for TSR = 4.50.

Figure 10 presents temporal variations in lift and drag coefficients of single blades of VAWTs with respect to α , denoted as $C_{L}$ and $C_{D}$ , respectively. Here, the data for Blade 11 of dual-stage turbines was plotted for simplicity and brevity. To illustrate the effect of ϕ of the unsteady aerodynamics of these rotating blades, $C_{L}$ and $C_{D}$ for the VAWT with ϕ=60° were included as well. In Figure 10(a1), the rotating blades experienced less lift for α>2° . A static airfoil experiences stall at $\alpha_{\mathrm{ss}}=12{}^{\circ}$ , and the blades in the single-stage VAWT and dual-stage turbines attained their maximum $C_{L}$ for $\alpha >\alpha_{\mathrm{ss}}$ and the entire range of ϕ was considered here. The data for static foils was computed using XFOIL (Drela, 1989). These results also present that the blades of the single-stage turbine and dual-stage VAWT with ϕ=0° showed almost similar hysteresis. However, the dual-stage turbine blade with ϕ=60° exhibited larger hysteresis. These observations also held for TSR=3.50 in Figure 10(b1). For TSR=4.50 in Figure 10(c1), the blades did not undergo $\alpha >\alpha_{\mathrm{ss}}$ at any stage of the rotational cycle. It appears that flow remained attached to the blades for all α , but the pressure difference for their outer and inner surfaces was less than that required to have $C_{L}$ at any α . Moreover, the blades for these VAWTs experienced $C_{D}$ greater than that of a static airfoil for most ranges of α . Moreover, dual-stage turbine blades showed larger hysteresis for both ϕ compared to that of the single-stage VAWT.

**Figure 11.**
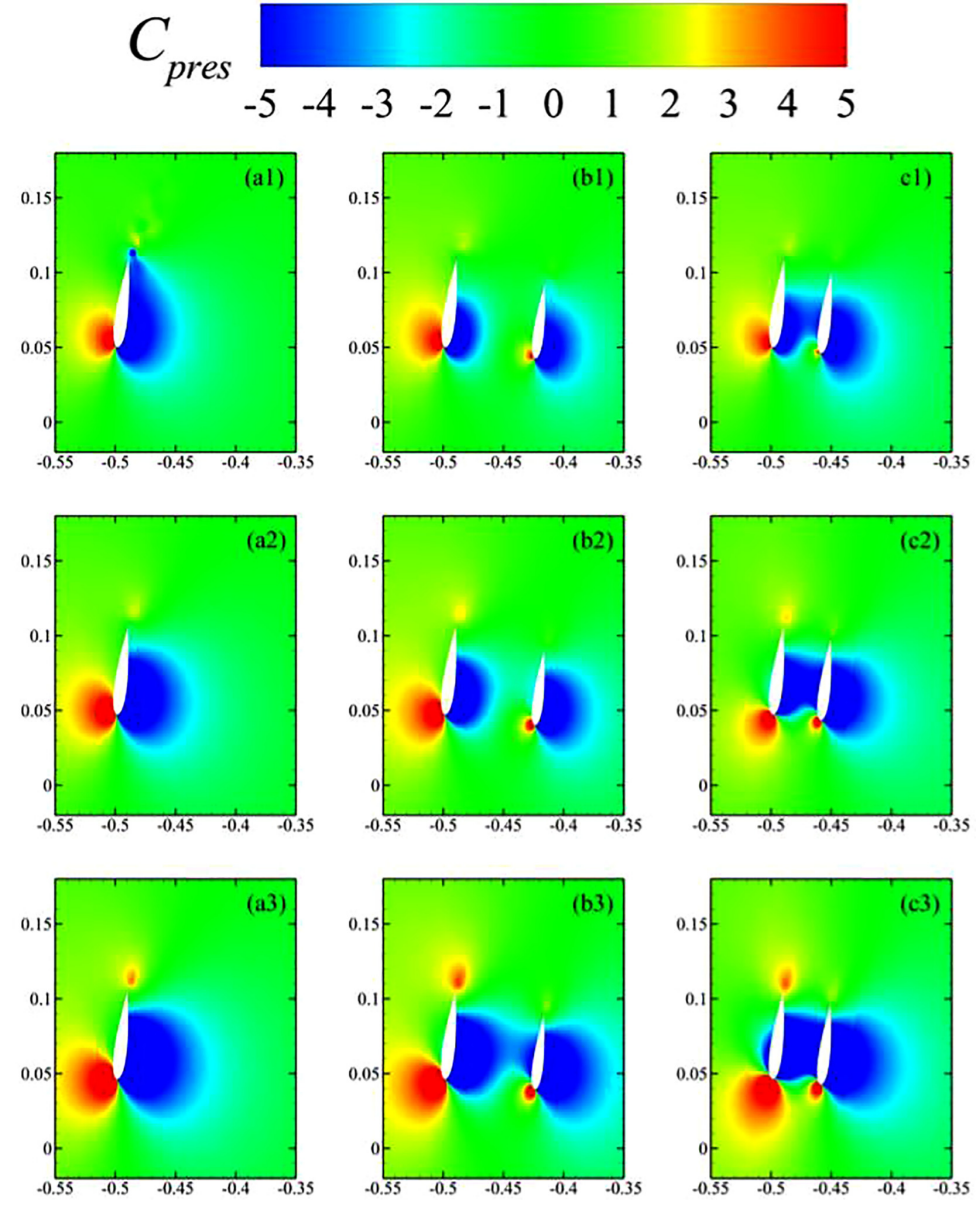
Contours of $C_{pres}$ around the blades of a single-stage turbine (left column), dual-stage turbine with ϕ=0° & $R_{2}/R_{1}=0.85$ (middle column), and dual-stage turbine with ϕ=0° & $R_{2}/R_{1}=0.92$ (right column) positioned at ϕ=81° , where the top, middle, and bottom rows correspond to TSR = 2.50, 3.50, and 4.50, respectively.

These trends in dynamic loads were then related with pressure variations around the blades through contours of the pressure coefficient ( $C_{pres}$ ) in Figure 11. For TSR=2.50 (Figure 11(a1)–(c1)), there appeared to be a large difference in the distributions of $C_{p}$ around the blades for single- and double-stage VAWTs. A low-pressure region expanded over the whole inner surface of the blade of the single-stage turbine, which was contracted due to the presence of the inner rotor in dual-stage turbines. These pressure distributions also explained the lower power production by the inner rotors. Outer surfaces of its blades did not have significantly higher-pressure regions formed over them, which was more prominent for the dual-stage turbine with $R_{2}/R_{1}=0.92$ . At higher TSRs , both high- and low-pressure regions expanded for the blade of a single-stage turbine (see Figure 11(a2) and (a3)). It contributed to produce more lift by the blade that consequently improved its power production. Similar observations were made for blades of the dual-stage turbines as well in Figure 11(b2), (b3), (c2), and (c3). In Figure 11(c3), the formation of a low-pressure region expanded over almost half of the outer surface of the blade in the primary rotor. Perhaps, such phenomena were responsible for the sudden deterioration of its aerodynamic performance for higher TSRs shown in Figure 8.

Figure 10(a1) and (b1) show almost overlapping hysteresis curves for lift and drag coefficients for the single-rotor and the dual-rotor VAWTs with $R_{2}/R_{1}=0.85$ . In contrast, the dual-rotor VAWT with $R_{2}/R_{1}=0.92$ starts showing differences from TSR=2.5 , as exhibited in Figure 10(a1). It may be explained by the interaction of low-pressure zones of the inner airfoils with the outer one. It is until TSR=3.5 that the low-pressure zone of the inner airfoil of dual-rotor VAWT with $R_{2}/R_{1}=0.85$ does not extend to the outer airfoil, unlike the case with $R_{2}/R_{1}=0.95$ that reaches the outer airfoil, starting from TSR=2.5 in Figure 11(c1). Figure 11(b3) shows how the low-pressure zone of the inner airfoil does reach the outer airfoil that is similar to the scenarios shown in Figure 11(c1)–(c3). Please note that the dual-rotor VAWT with $R_{2}/R_{1}=0.92$ exhibits this interaction from TSR=2.5 since both rotors are very closely placed.

**Figure 12.**
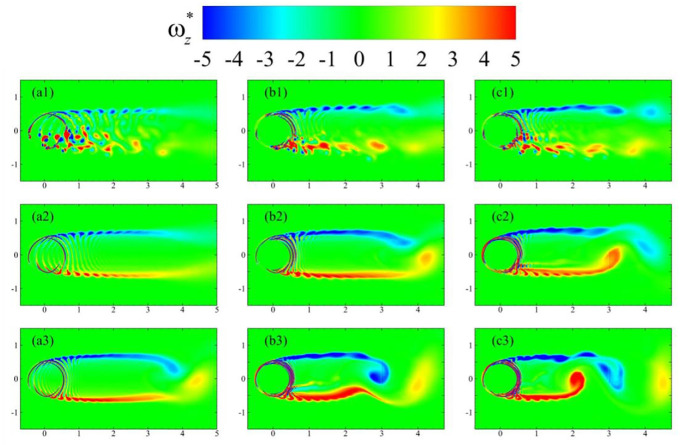
Contours of nondimensional vorticity ( $\omega_{z}$ ) around the blades of a single-stage turbine (left column, a1, a2, & a3), dual stage turbine with ϕ=0° & $R_{2}/R_{1}=0.85$ (middle column, b1, b2, & b3), and dual-stage turbine with ϕ=0° & $R_{2}/R_{1}=0.92$ (right column, c1, c2, & c3) positioned at ϕ=81° , whereas the top, middle, and bottom rows correspond to TSR = 2.50, 3.50, and 4.50, respectively.

Finally, the details of overall wake formations behind single- and dual-stage turbines are shown in Figure 12. Figure 12(a1), (b1), and (c1) show vorticity contours of the flow around a single-stage turbine, and double-stage VAWTs with $R_{2}/R_{1}=0.85$ and $R_{2}/R_{1}=0.92$ , respectively. There existed discrete vortices shed by the foil at θ=0° for the single-stage VAWT. Near the opposite end of the turbine, complex vortical structures were present, which indicated extensive vortex-blade interactions. Such phenomena were more noticeable for 90°<θ<270° . For dual-stage turbines, the vortices were more interconnected with each other with enhanced vorticity levels in the respective wakes due to the additional contribution by the inner-stage rotors. The upper vortex array was thickened for the dual-stage VAWT with the greater ratio of radii. This observation hinted at more constructive interactions (Khalid et al., 2021) between the vortices shed by primary and secondary blades in this case. This may also be the primary reason for higher drag production at this TSR possibly resulting in more torque experienced by the turbine (see Figure 8). At higher TSRs , the wake of turbines resembled those of circular cylinders of large diameters, which are shown in the plots of middle and bottom rows of Figure 12. However, the shedding of big vortices, with length scales of almost the order of the radii of turbines, was more prominent for dual-stage VAWTs. Under these conditions, the shear layer starts rolling at a distance of 3.50D and 3D from their centers in Figure 12(b2) and (c2), respectively, for turbines rotating with TSR=3.50 . This formation of big vortices not only enhanced drag production for these conditions, but also it would affect the performance of any turbines installed downstream. For even higher TSRs , such as the one in Figure 12(a3), the single-stage turbine also showed the signs of this vortex shedding at a distance of 4D from its center, which may be related to decrements in the performance of the single-stage turbines, as shown in Figure 6(a). For dual-stage VAWTs in Figure 12(b3) and 12(c3), this shedding process was more intense and started in the regions closer to the turbines. This also contributed to the reduced performance of these turbines at higher tip-speed ratios.

The low-pressure zone interaction, shown in Figure 11, is demonstrated in the merging of the vortices shown in Figure 12 in the middle and bottom rows produced by the foils. It is from TSR=4.5 in Figure 12(b3) that vortices are merged for the dual-rotor VAWT with $R_{2}/R_{1}=0.85$ . For TSR<4.5 , vortices are separated from each other as developed similarly to the unique vortex that the single-rotor VAWT presents.

## Conclusions

The flow around single- and dual-stage vertical-axis wind turbines were numerically examined for TSR ranging from 1.50 to 4.50 . It was determined that dual-stage VAWTs outperformed their single-stage counterparts by enhancing power production manifolds at 1.50<TSRs<3.0 . However, their performance substantially degraded for higher TSRs . The geometric phase angle between the blades in two rotors of dual-stage turbines did not significantly impact their performance in a time-averaged sense. However, bringing these rotors closer had a greater impact on their power production. This change enhanced their $C_{P}$ from 400% to 600% at TSR=2.0 . The analysis of underlying flow mechanisms associated with trends of performance parameters of these turbines revealed contributing factors for degraded power generation of dual-stage turbines at higher tip-speed ratios: (i) the formation of low-pressure regions over the outer surfaces of the blades of primary rotors, which adversely affects their lift production; (ii) the rolling of shear layers in the wake of these turbines and forming and shedding of large vortices. These coherent structures with length scales of the order of radii of these VAWTs generated greater drag. Further investigations are required to examine the impact of other important parameters to better understand the power production performance and self-starting capabilities of dual-stage turbines. The metrics presented here, indicate that the proposed nature-inspired designs are expected to improve their utilization in urban environments, where the TSR is usually lower. These systems also provide substantial improvements at high TSRs, which are likely in open fields or rural areas. However, one still needs to address how these multi-rotor VAWT designs behave in highly turbulent flows around buildings or complex terrains. Our future studies will address these important questions to enable the commercialization of nature-inspired designs.

## References

[bibr1-0309524X231212638] ANSYS (2020) Ansys Fluent 2020r2. Theory Guide. Canonsburg, PA: ANSYS, Inc.

[bibr2-0309524X231212638] Arpino F Cortellessa G Dell’Isola M , et al. (2017) Cfd simulations of power coefficients for an innovative Darrieus style vertical axis wind turbine with auxiliary straight blades. Journal of Physics Conference Series 923: 012036.

[bibr3-0309524X231212638] Arpino F Cortellessa G Massarotti N , et al. (2020) Numerical performance assessment of a novel Darrieus-style vawt with auxiliary straight blades. Journal of Physics Conference Series 1589: 012020.

[bibr4-0309524X231212638] Arpino F Scungio M Cortellessa G (2018) Numerical performance assessment of an innovative Darrieus-style vertical axis wind turbine with auxiliary straight blades. Energy Conversion and Management 171: 769–777.

[bibr5-0309524X231212638] Asr MT Nezhad EZ Mustapha F , et al. (2016) Study on start-up characteristics of H-Darrieus vertical axis wind turbines comprising NACA 4-digit series blade airfoils. Energy 112: 528–537.

[bibr6-0309524X231212638] Bazilevs Y Korobenko A Deng X , et al. (2014) Fluid–structure interaction modeling of vertical-axis wind turbines. Journal of Applied Mechanics 81(8): 081006.

[bibr7-0309524X231212638] Bhuyan S Biswas A (2014) Investigations on self-starting and performance characteristics of simple H and hybrid H-Savonius vertical axis wind rotors. Energy Conversion and Management 87: 859–867.

[bibr8-0309524X231212638] Brownstein ID Kinzel M Dabiri JO (2016) Performance enhancement of downstream vertical-axis wind turbines. Journal of Renewable and Sustainable Energy 8(5): 053306.

[bibr9-0309524X231212638] Castelli MR Englaro A Benini E (2011) The Darrieus wind turbine: Proposal for a new performance prediction model based on CFD. Energy 36: 4919–4934.

[bibr10-0309524X231212638] Dabiri JO (2011) Potential order-of-magnitude enhancement of wind farm power density via counter-rotating vertical-axis wind turbine arrays. Journal of Renewable and Sustainable Energy 3(4): 043104.

[bibr11-0309524X231212638] Didane DH Rosly N Zulkafli MF , et al. (2018) Performance evaluation of a novel vertical axis wind turbine with coaxial contra-rotating concept. Renewable Energy 115: 353–361.

[bibr12-0309524X231212638] Didane DH Rosly N Zulkafli MF , et al. (2019) Numerical investigation of a novel contra-rotating vertical axis wind turbine. Sustainable Energy Technologies and Assessments 31: 43–53.

[bibr13-0309524X231212638] Drela M (1989) Xfoil: An analysis and design system for low Reynolds number airfoils. In: Low reynolds number aerodynamics: Proceedings of the conference, Notre Dame, Indiana, USA, 5–7 June 1989, pp. 1–12. Berlin, Heidelberg: Springer Berlin Heidelberg,

[bibr14-0309524X231212638] Eriksson S Bernhoff H Leijon M (2008) Evaluation of different turbine concepts for wind power. Renewable and Sustainable Energy Reviews 12(5): 1419–1434.

[bibr15-0309524X231212638] Galinos C Larsen TJ Madsen HA , et al. (2016) Vertical axis wind turbine design load cases investigation and comparison with horizontal axis wind turbine. Energy Procedia 94: 319–328.

[bibr16-0309524X231212638] Ghasemian M Nejat A (2015) Aero-acoustics prediction of a vertical axis wind turbine using large eddy simulation and acoustic analogy. Energy 88: 711–717.

[bibr17-0309524X231212638] Ghosh A Biswas A Sharma KK , et al. (2015) Computational analysis of flow physics of a combined three bladed Darrieus Savonius wind rotor. Journal of the Energy Institute 88(4): 425–437.

[bibr18-0309524X231212638] Gungor A Hemmati A (2020) Wake symmetry impacts the performance of tandem hydrofoils during in-phase and out-of-phase oscillations differently. Physical Review E 102(4–1): 043104. 10.1103/PhysRevE.102.043104 33212661

[bibr19-0309524X231212638] Gungor A Hemmati A (2021) Implications of changing synchronization in propulsive performance of side-by-side pitching foils. Bioinspiration & Biomimetics 16(3): 036006. 10.1088/1748-3190/abe54b 33571986

[bibr20-0309524X231212638] Hassanpour M Azadani LN (2021) Aerodynamic optimization of the configuration of a pair of vertical axis wind turbines. Energy Conversion and Management 238: 114069.

[bibr21-0309524X231212638] Hill N Dominy R Ingram G , et al. (2009) Darrieus turbines: The physics of self-starting. Proceedings of the Institution of Mechanical Engineers Part A Journal of Power and Energy 223(1): 21–29.

[bibr22-0309524X231212638] Khalid MSU Akhtar I Dong H (2016) Hydrodynamics of a tandem fish school with asynchronous undulation of individuals. Journal of Fluids and Structures 66: 19–35.

[bibr23-0309524X231212638] Khalid MSU Wang J Akhtar I , et al. (2021) Why do anguilliform swimmers perform undulation with wavelengths shorter than their bodylengths? Physics of Fluids 33(3): 031911.

[bibr24-0309524X231212638] Khalid MSU Wood D Hemmati A (2022) Self-starting characteristics and flow-induced rotation of single- and dual-stage vertical-axis wind turbines. Energies 15(24): 9365.

[bibr25-0309524X231212638] Kinsey T Dumas G (2012) Optimal tandem configuration for oscillating-foils hydrokinetic turbine. Journal of Fluids Engineering 134(3): 031103.

[bibr26-0309524X231212638] Kinzel M Mulligan Q Dabiri JO (2012) Energy exchange in an array of vertical-axis wind turbines. Journal of Turbulence 13(1): N38.

[bibr27-0309524X231212638] Kumbernuss J Chen J Yang HX , et al. (2012) Investigation into the relationship of the overlap ratio and shift angle of double stage three bladed vertical axis wind turbine (VAWT). Journal of Wind Engineering and Industrial Aerodynamics 107–108: 57–75.

[bibr28-0309524X231212638] Lam HF Peng HY (2017a) Measurements of the wake characteristics of co- and counter-rotating twin H-rotor vertical axis wind turbines. Energy 131: 13–26.

[bibr29-0309524X231212638] Lam HF Peng HY (2017b) Development of a wake model for Darrieus-type straight-bladed vertical axis wind turbines and its application to micro-siting problems. Renewable Energy 114: 830–842.

[bibr30-0309524X231212638] Li S Li Y Yang C , et al. (2021) Experimental investigation of solidity and other characteristics on dual vertical axis wind turbines in an urban environment. Energy Conversion and Management 229: 113689.

[bibr31-0309524X231212638] Malael I Dragan V (2018) Numerical and experimental efficiency evaluation of a counter-rotating vertical axis wind turbine. Engineering, Technology and Applied Science Research 8(4): 3282–3286.

[bibr32-0309524X231212638] Menter FR (1994) Two-equation eddy-viscosity turbulence models for engineering applications. AIAA Journal 32(8): 1598–1605.

[bibr33-0309524X231212638] Mohamed MH (2014) Aero-acoustics noise evaluation of H-rotor darrieus wind turbines. Energy 65: 596–604.

[bibr34-0309524X231212638] Mohamed MH (2016) Reduction of the generated aero-acoustics noise of a vertical axis wind turbine using CFD (computational fluid dynamics) techniques. Energy 96: 531–544.

[bibr35-0309524X231212638] Naccache G Paraschivoiu M (2018) Parametric study of the dual vertical axis wind turbine using CFD. Journal of Wind Engineering and Industrial Aerodynamics 172: 244–255.

[bibr36-0309524X231212638] Ni L Miao W Li C , et al. (2021) Impacts of gurney flap and solidity on the aerodynamic performance of vertical axis wind turbines in array configurations. Energy 215: 118915.

[bibr37-0309524X231212638] Pan Y Dong H (2020) Computational analysis of hydrodynamic interactions in a high-density fish school. Physics of Fluids 32(12): 121901.

[bibr38-0309524X231212638] Peng HY Han ZD Liu HJ , et al. (2020) Assessment and optimization of the power performance of twin vertical axis wind turbines via numerical simulations. Renewable Energy 147: 43–54.

[bibr39-0309524X231212638] Rezaeiha A Kalkman I Blocken B (2017a) CFD simulation of a vertical axis wind turbine operating at a moderate tip speed ratio: Guidelines for minimum domain size and azimuthal increment. Energy 107: 373–385.

[bibr40-0309524X231212638] Rezaeiha A Kalkman I Blocken B (2017b) Effect of pitch angle on power performance and aerodynamics of a vertical axis wind turbine. Applied Energy 197: 132–150.

[bibr41-0309524X231212638] Rezaeiha A Montazeri H Blocken B (2019) On the accuracy of turbulence models for CFD simulations of vertical axis wind turbines. Energy 180: 838–857.

[bibr42-0309524X231212638] Saad AS Elwardany A El-Sharkawy II , et al. (2021) Performance evaluation of a novel vertical axis wind turbine using twisted blades in multi-stage savonius rotors. Energy Conversion and Management 235: 114013.

[bibr43-0309524X231212638] Sahebzadeh S Rezaeiha A Montazeri H (2020) Towards optimal layout design of vertical-axis wind-turbine farms: Double rotor arrangements. Energy Conversion and Management 226: 113527.

[bibr44-0309524X231212638] Scungio M Arpino F Focanti V , et al. (2016) Wind tunnel testing of scaled models of a newly developed Darrieus-style vertical axis wind turbine with auxiliary straight blades. Energy Conversion and Management 130: 60–70.

[bibr45-0309524X231212638] Siddiqui MS Durrani N Akhtar I (2015) Quantification of the effects of geometric approximations on the performance of a vertical axis wind turbine. Renewable Energy 74: 661–670.

[bibr46-0309524X231212638] Su H Dou B Qu T , et al. (2020) Experimental investigation of a novel vertical axis wind turbine with pitching and self-starting function. Energy Conversion and Management 217: 113012.

[bibr47-0309524X231212638] Sun X Zhu J Li Z , et al. (2021) Rotation improvement of vertical axis wind turbine by offsetting pitching angles and changing blade numbers. Energy 215: 119177.

[bibr48-0309524X231212638] Tahani M Razavi M Mirhosseini M , et al. (2020) Unsteady aerodynamic performance of dual-row H-darrieus vertical axis wind turbine. Energy Equipment and Systems 8(1): 55–80.

[bibr49-0309524X231212638] Thé J Yu H (2017) A critical review on the simulations of wind turbine aerodynamics focusing on hybrid RANS-LES methods. Energy 138: 257–289.

[bibr50-0309524X231212638] Torabi Asr M Osloob R Mustapha F , et al. (2016) Double-stage h-darrieus wind turbine-rotor aerodynamics. Applied Mechanics and Materials 829: 21–26.

[bibr51-0309524X231212638] Vergaerde A De Troyer T Muggiasca S , et al. (2020) Experimental characterisation of the wake behind paired vertical-axis wind turbines. Journal of Wind Engineering and Industrial Aerodynamics 206: 104353.

[bibr52-0309524X231212638] Zanforlin S Nishino T (2016) Fluid dynamic mechanisms of enhanced power generation by closely spaced vertical axis wind turbines. Renewable Energy 99: 1213–1226.

[bibr53-0309524X231212638] ZSB-AB (2011) Doppelter Darrieus-rotor. DE202011002702U, May.

